# Editorial: Biomolecular modifications in endocrine-related cancers, volume II

**DOI:** 10.3389/fendo.2024.1485789

**Published:** 2024-10-02

**Authors:** Xianquan Zhan, Na Li, Godfrey Grech

**Affiliations:** ^1^ Shandong Provincial Key Laboratory of Precision Oncology, Shandong Provincial Key Medical and Health Laboratory of Ovarian Cancer Multiomics, Jinan, Shandong, China; ^2^ Jinan Key Laboratory of Cancer Multiomics, Shandong Cancer Hospital and Institute, Shandong First Medical University, Jinan, Shandong, China; ^3^ Department of Pathology, University of Malta, Msida, Malta

**Keywords:** post-translational modification, post-transcriptional modification, DNA modification, proteoform, proteoformics, genomics, transcriptomics, proteomics

**Figure 1:**
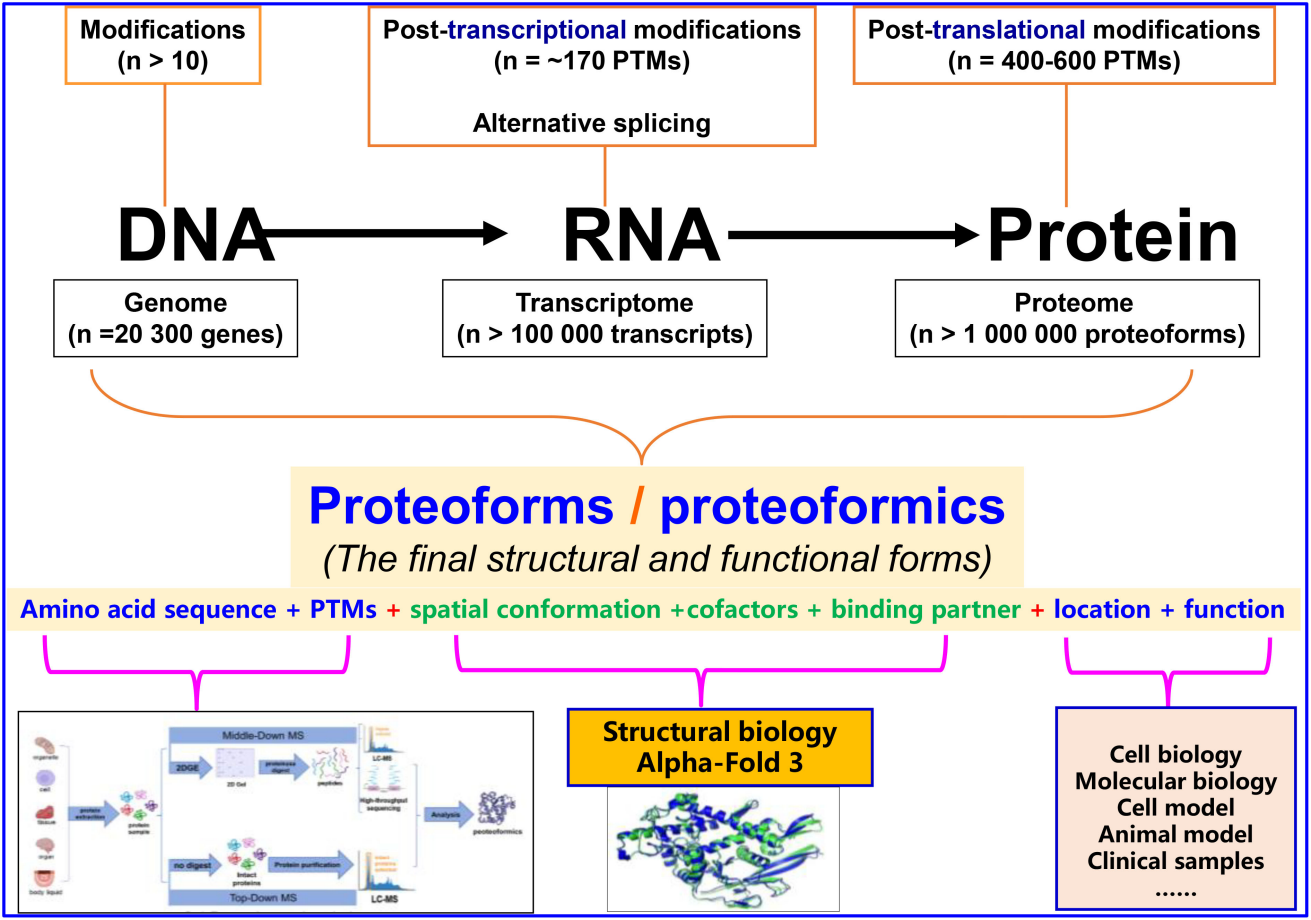
Effects of biomolecular modifications on proteoforms, proteoform concept, and proteoformics methodology. Modified from Zhan X et al. (8) with copyright permission from Springer Nature. Modified from Li, Desiderio, and Zhan (9) with copyright permission from Wiley, and from Zhan, Long, and Lu (10) with copyright permission from Elsevier.

Following the successful publication of the first volume on modifications in biomolecules (proteins, RNAs, and DNAs) (https://www.frontiersin.org/research-topics/24966/biomolecular-modifications-in-endocrine-related-cancers) and the rapid progress in this field, we have edited a second volume to further highlight the importance and complexity of biomolecular (proteins, RNAs, and DNAs) modifications in the cancer biological system. First, we focused on genomics, transcriptomics, proteomics, and bioinformatics, which enable in-depth research on large-scale biomolecular modifications, including the determination of modification sites and abundances in DNAs, RNAs, and proteins, modification-mediated signaling pathways and functions, and antagonistic and synergistic effects between modifications in a biomolecule (1). Second, we focused on in DNAs, RNAs, and proteins, which are very important factors in causing the diversity of a biomolecule, ultimately resulting in proteoforms that are comprehensively determined by the amino acid sequence, post-translational modifications (PTMs), spatial conformation, cofactors, binding partners, location, and a function (2, 3). Proteoforms are the final forms of the structures and functions of a gene or protein, and are the basic components of a proteome. Proteoformics involves the study of both targeted proteoforms based on a given gene and non-targeted proteoforms based on a given disease or pathophysiological condition. The study of both is based on mass spectrometry (MS) to determine the amino acid sequence and PTMs of a proteoform. The development of proteoformics will greatly advance the in-depth study of biomolecular modifications (**Figure 1**) (1, 4, 5). The MS-based proteoformics mainly include two approaches, namely Top-Down MS and Middle-Down MS (2DE-LC/MS). Top-Down MS has a high ion inhibitory effect, and works well for <30 kDa proteoforms, which only covers a sub-proteome. For 2DE-LC/MS, 2DE is the high-throughput separation technique, and LC-MS/MS has a low ion inhibitory effect; therefore, 2DE-LC/MS works well for all molecular-weight proteoforms, covering the entire proteome. Third, we focused on the rapidly developing AlphaFold 3AI technology in the field of structural biology (6, 7), which can be used to accurately resolve the structures and intermolecular interactions of large-scale proteoforms in a high-throughput manner, revealing the effects of biomolecular modifications on the structure and function of biomolecules in a given biological system (**Figure 1**). Compared to the low-throughput classical structural biology techniques such as X-ray diffraction and cryo-electron microscopy (Cryo-EM), AlphaFold 3 will greatly advance the high-throughput study of proteoforms and the role of post-translational modifications (PTMs). Fourth, we considered that all cancers are endocrine-related, and biomolecular modifications are associated with the onset and development of cancers. Biomolecular modifications are an important way to thoroughly address the molecular mechanisms of cancer and to find novel biomarkers and therapeutic targets for endocrine-related cancers (1). The present Research Topic includes the contributions of molecular modifications to therapeutic targets and biomarkers of endocrine-related cancers, which will establish a platform to stimulate researchers for broad, in-depth, and systemic studies on biomolecular modifications in cancers.

This Research Topic includes 5 articles. (i) The first study discusses the contribution of protein ubiquitination to carcinogenesis and the discovery of targeted drugs for lung cancer (Ye et al.). This indicates that (a) the ubiquitin-proteasome pathway is involved in protein degradation in addition to regulating other metabolic pathways. (b) Targeting the ubiquitin-proteasome pathway system for cancer therapy provides a new perspective for developing new anti-cancer drugs for lung cancer. (c) Targeted degradation technology is a mediator to link ubiquitin ligases to target proteins, which is a new strategy to develop new anti-cancer drugs for lung cancer. (ii) The second article addresses the relationship between CXC chemokine-receptor 4 (CXCR4) and gastroenteropancreatic neuroendocrine neoplasms grade 3 (GEP-NENs G3), and its clinical and prognostic value (Pang et al.). Modifications may mediate the differential expression of CXCR4 to act as a new immunohistochemical biomarker for GEP-NENs G3 diagnosis. (iii) The third article addresses the prognostic signature of nonspecific mucinous adenocholangiocarcinoma based on a retrospective survey of 22,509 patients (Azhar et al.), which found that the number of primary tumors, tumor size, age, lymph node status, grade, AJCC stage, metastases, chemotherapy, and surgery act as independent prognostic parameters for patients with mucinous and ordinary cholangiocarcinoma. The authors also established a nomogram that is important in the overall clinical management of cholangiocarcinoma. These phenotypes in the prognostic model are associated with different types of biomolecular modifications. (iv) The fourth article is a case report of a Colombian family carrying a novel variant of multiple endocrine neoplasia type 1 (MEN1) associated with a rare ACTH-producing pancreatic neuroendocrine tumor (Riaño-Moreno et al.), demonstrating a new subtype of ACTH-producing pancreatic neuroendocrine tumor in the spectrum of MEN1 variants. (v) The fifth article provides new insights into the multiple hormonal regulatory systems in thyroid cancer beyond the hypothalamic–pituitary–thyroid axis (Chen et al.), including the effects of estrogen, thyroid hormone, thyroid stimulating hormone, growth hormone (GH), androgen, insulin-like growth factor-1 (IGF-1), and glucocorticoid in the thyroid cancer hormonal system. Numerous studies have clearly demonstrated that biomolecular changes are involved in the regulation of these hormones.

As the above summary clearly demonstrates, biomolecular modifications are of crucial importance in the listed cancers. However, biomolecular changes have not been sufficiently studied in the context of cancer, where they are involved in every aspect of predictive, preventive, and personalized medicine (3P medicine) (11–24). We strongly believe that proteoformics will play an important role in clarifying the minute changes in the structures and functions of a biomolecule, which will promote effective biomarker discovery for in-depth insights into the molecular mechanisms of cancer, identify reliable therapeutic targets and drugs for cancer therapy, and construct tumor biomarkers for accurate prediction, diagnosis, and prognosis of cancer. However, it must be acknowledged that this Research Topic only collects studies on a few biomolecular modifications related to endocrine-related cancers, thereby acting as a catalyst to stimulate and encourage more researchers to conduct biomolecular modification studies in the future. Once again, we emphasize that proteoformics studies based on molecular modifications will provide a bright future for the treatment of endocrine-related cancers in 3P medicine and precision medicine practice.
